# Supplementary material for: Integrated Analysis of Proteomics and Metabolomics for Heat Stress in Chinese Holstein Cows
Source: Animals (Basel). 2025 Oct 20;15(20):3049. doi: 10.3390/ani15203049 (PMC12562201; doi:10.3390/ani15203049)
Supplement: Supplementary file 1 [file animals-15-03049-s001.zip › animals-3878666-supplementary.pdf]

Table S1. The statistical information of protein quantification results.

**Samples**	**P1**	**P2**	**P3**	**P4**	**P5**	**P6**	**P11**	**P12**	**P13**	**P14**	**P15**	**P16**
Concentration (μg/μl)	5.50	6.30	5.60	5.50	5.50	5.20	4.40	5.40	4.60	5.80	4.70	6.30
Volum(μl)	200	200	200	200	200	200	200	200	200	200	200	200
Total Amount(μg)	1100	1260	1120	1100	1100	1040	880	1080	920	1160	940	1260
Sample evaluation	a	a	a	a	a	a	a	a	a	a	a	a

Table. S2 Liquid phase gradient of chromatographic column

**Time(min)**	**Buffer B gradient**
0 ~ 25	0
25 ~ 30	0% ~ 7%
30 ~ 65	7% ~ 40%
65 ~ 70	40% ~ 100%
70 ~ 85	1

Table S3. The primers of qRT-PCR for genes related to heat tolerance and the designed siRNAs for actinin alpha 4 gene (*ACTN4*) gene.

**Primer name**	**Primer sequence（5’-3’）**
ACTN4-F	CCCGACGAGAAGGCCATAAT
ACTN4-R	TGCTCGTTCTCCTGGTTGAC
HSP70-F	AGAAGAAGGTGCTGGACAAGT
HSP70-R	CTGGTACAGTCTGCTGATGATG
HSP27-F	CGTCAAGGTGGTGGACAAC
HSP27-R	TCGGATGAGACAGTGGACAC
caspase3-F	GAACTGGACTGTGGTATTGAGAC
caspase3-R	CGAGCCTGTGAGCGTACTT
Cytc-F	AGTGTGCCCAGTGCCATAC
Cytc-R	CTCCATCAGCGTCTCCTCTC
Bcl-2-F	TTCGCCGAGATGTCCAGTC
Bcl-2-R	GGTTGACGCTCTCCACACA
Bax-F	CGGAGATGAATTGGACAGTAACA
Bax-R	CAGTTGAAGTTGCCGTCAGAA
GAPDH-F	GAAGGTCGGAGTGAACGGAT
GAPDH-R	TTCTCTGCCTTGACTGTGCC
sh-1	GCAAGATGGTGTCGGACATCA
sh-2	GGAAGGATGGTCTTGCCTTCA
sh-3	GCAGTATGAGCGCAGCATTGT
NC	TTCTCCGAACGTGTCACGT

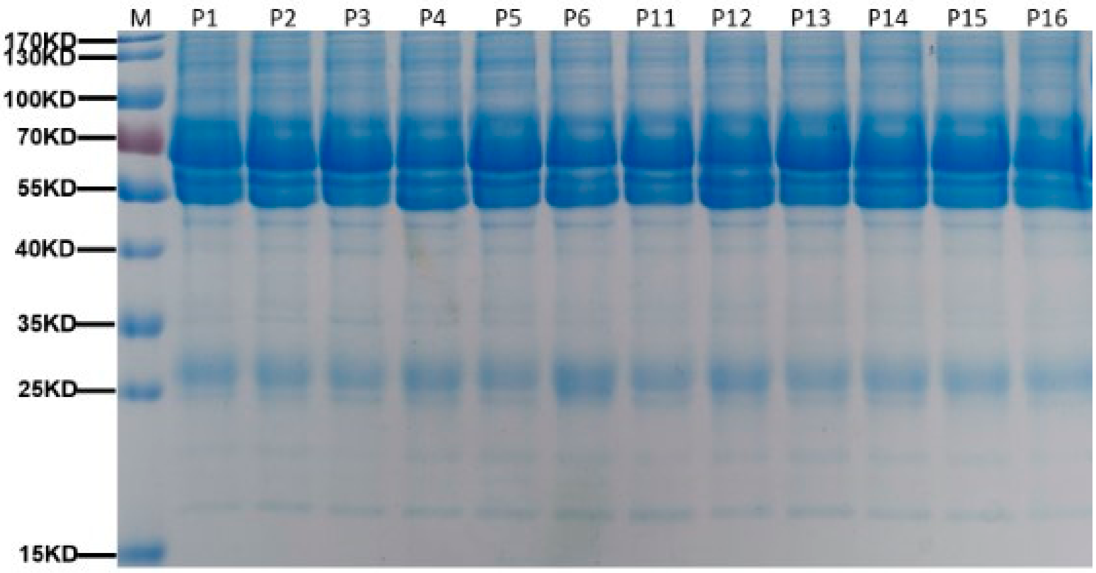

Figure S1. Detection of sodium dodecyl sulfate polyacrylamide gel electrophoresis (SDS-PAGE) gel.

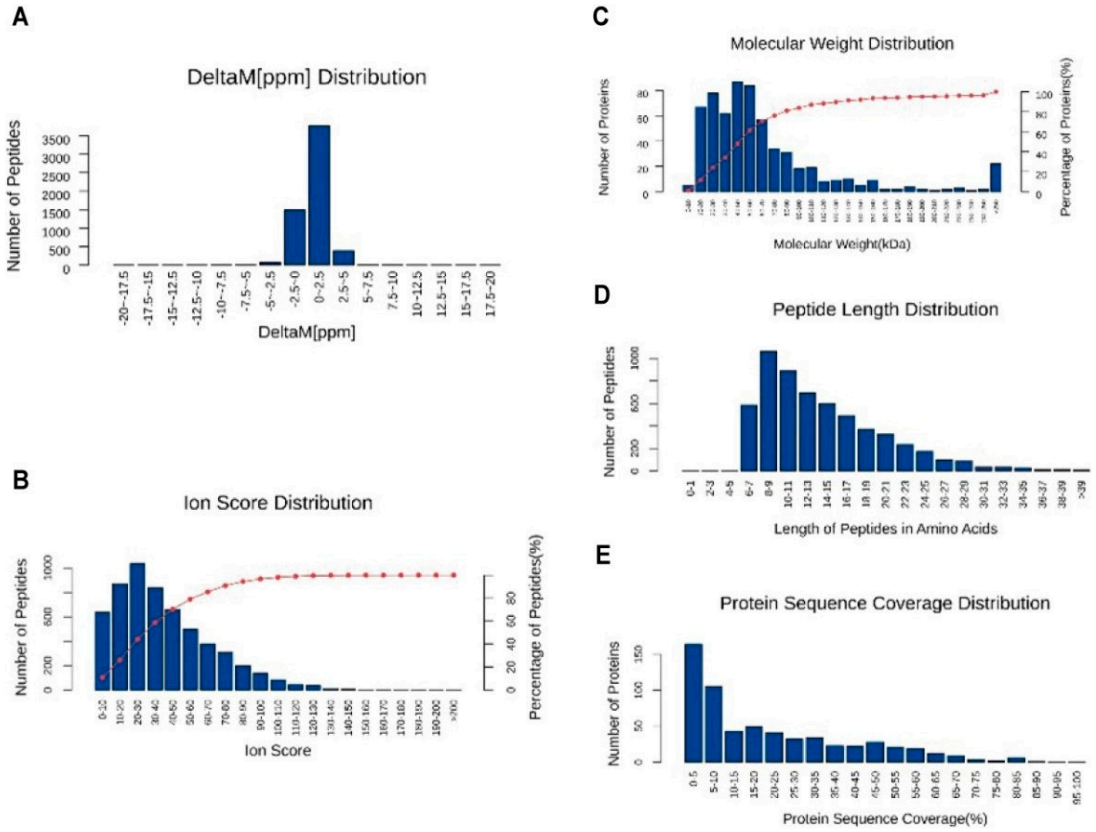

Figure S2. (A) Peptide mass deviation distribution. (B) Peptide ion score distribution. The X-axis indicated the peptide score detected by Mascot software; the main Y-axis indicated the number of identified peptides; the secondary Y-axis indicated the cumulative curve of the corresponding ion score. (C) Identification of relative molecular mass distribution of proteins. The X-axis indicated relative molecular mass of the identified protein; the main Y-axis indicated the protein number; the secondary Y-axis indicated the cumulative percentage of protein. (D) Peptide sequence length distribution. (E) Protein sequence coverage distribution.

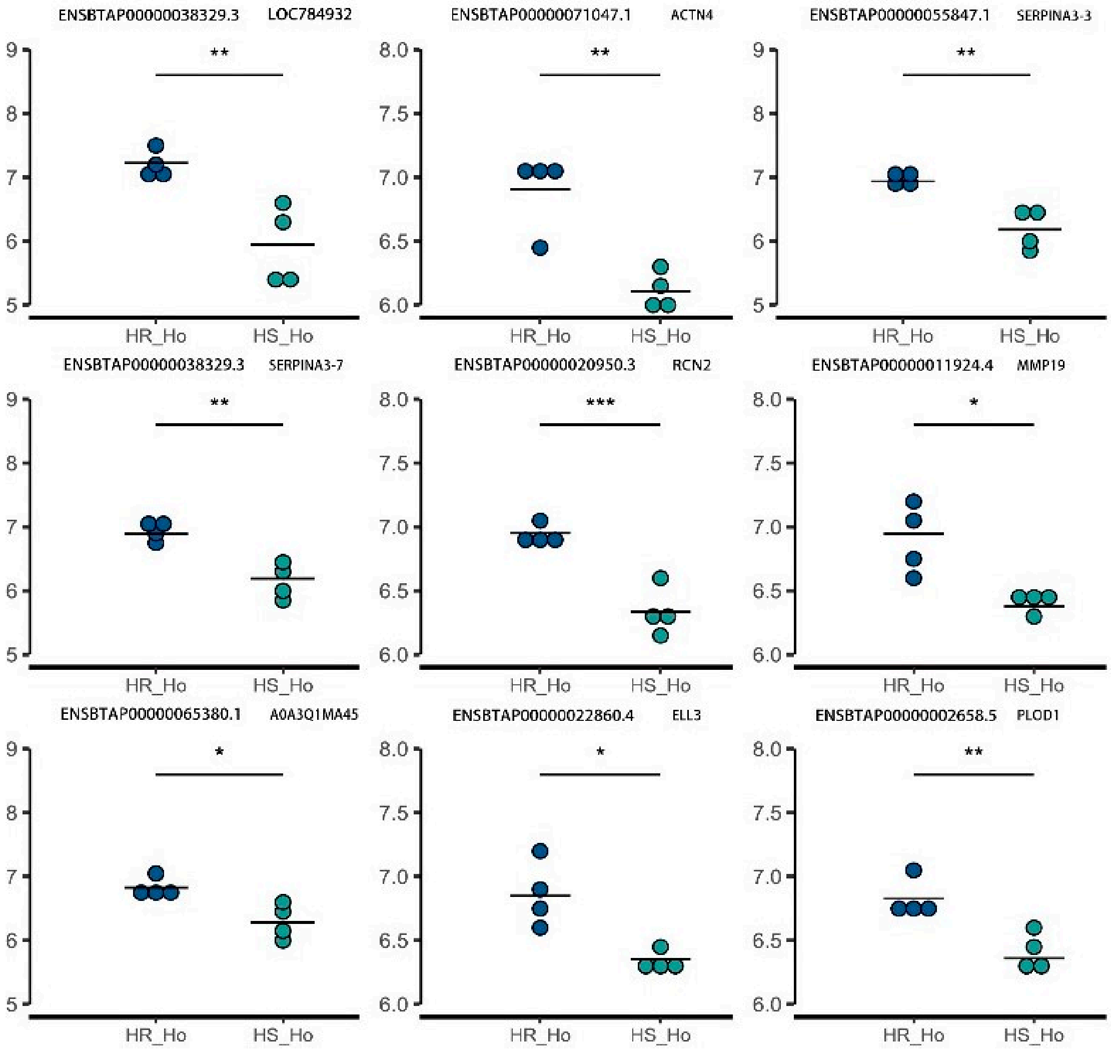

Figure S3. The top 9 proteins with the most up-regulation of expression levels in the heat-resistant (HR) group.

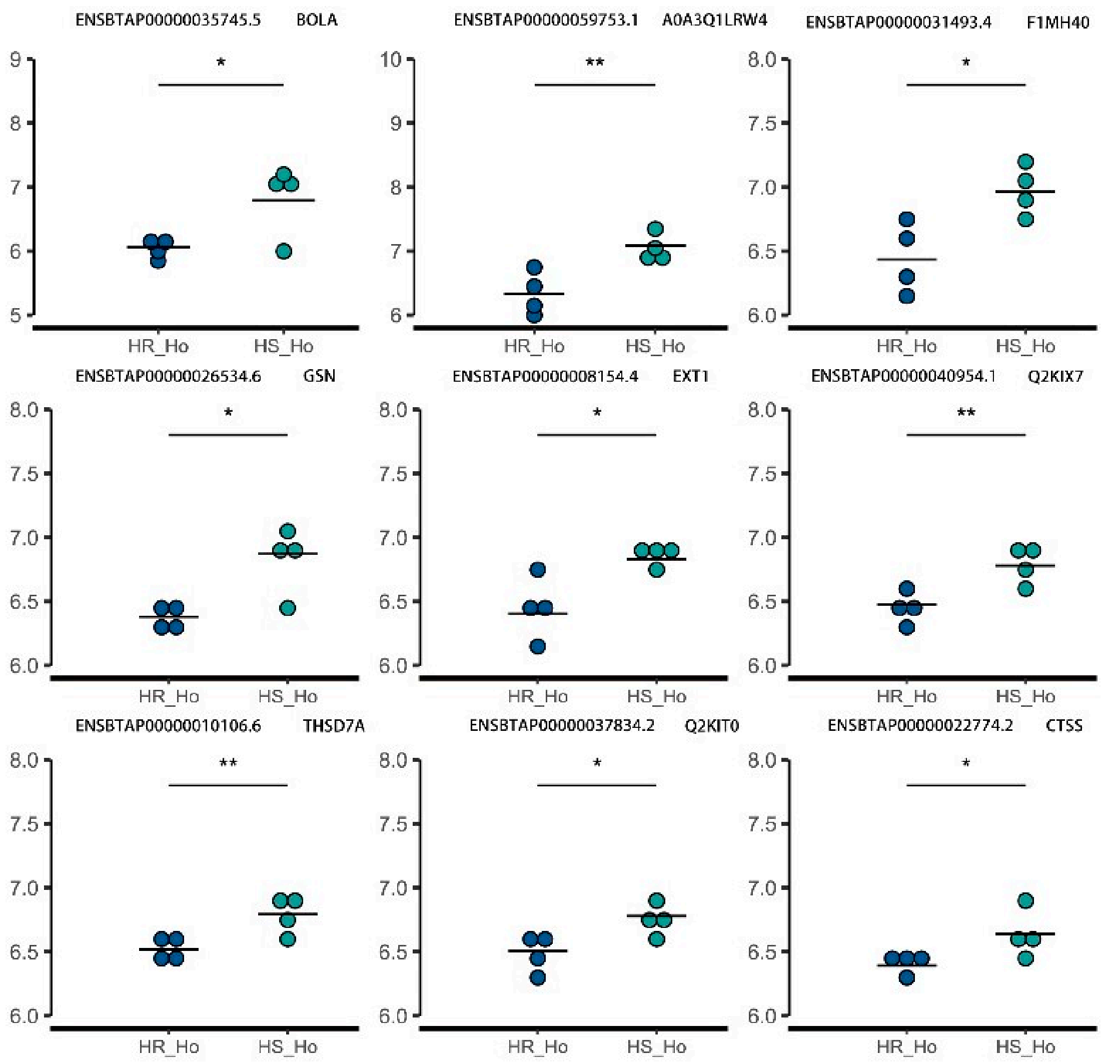

Figure S4. The top 9 proteins with the most up-regulation of expression levels in the heat-stressed (HS) group.

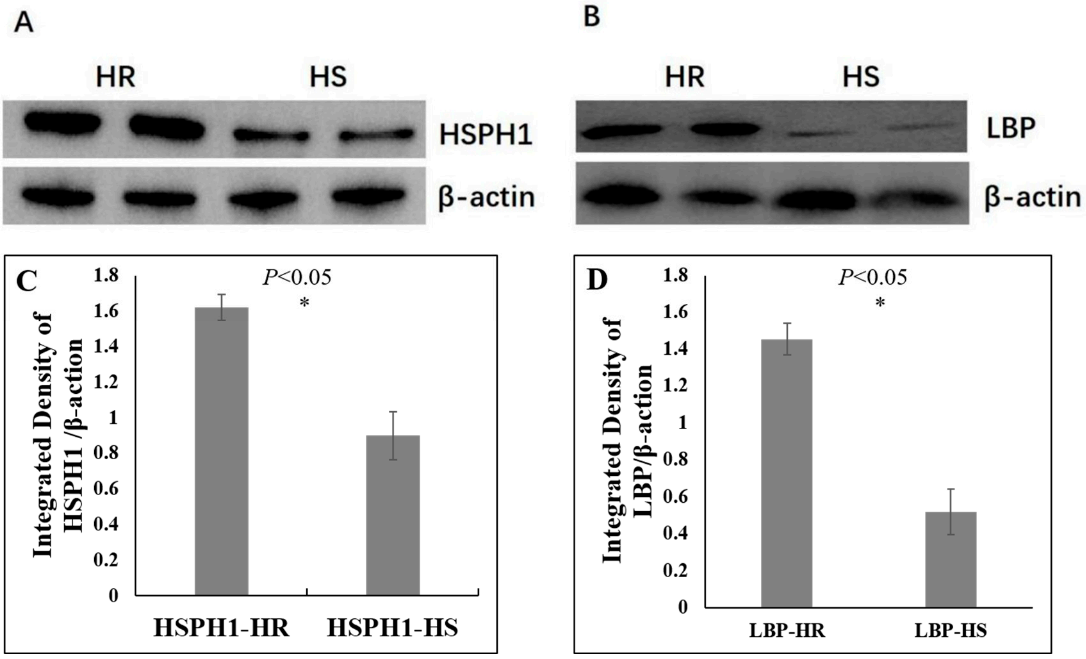

Figure S5. The western blot results of (A) HSPH1 and (B) LBP proteins. The statistical analysis for the integrated density of (C) HSPH1 and (D) LBP western blot result

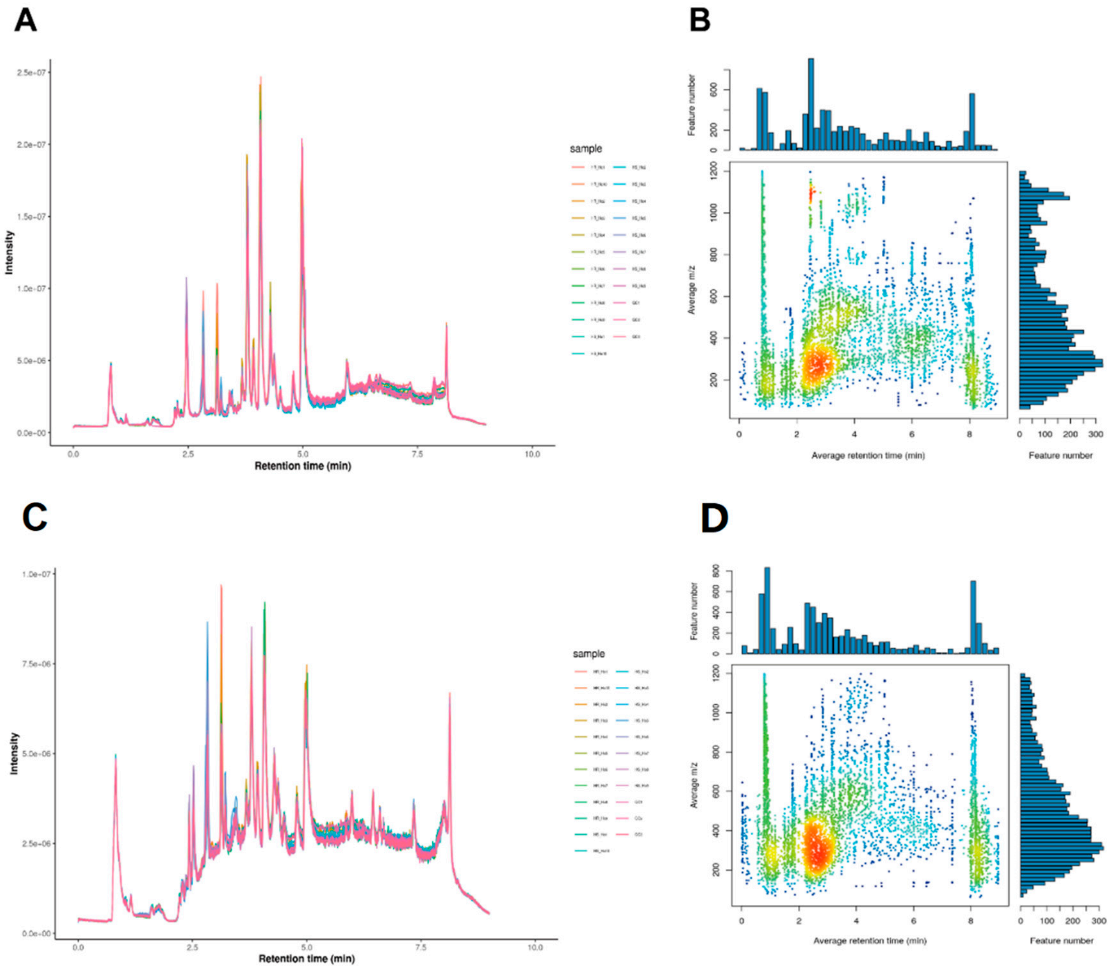

Figure S6. The total positive chromatograms (TIC) plots of the positive (A) and negative (C) metabolites with the overall mass spectrum signal intensity of all samples. The horizontal coordinate indicates the retention time point, and the vertical coordinate indicates the total strength of all ions in the mass spectrum. Each color represents a sample. The m/z-rt distribution map of the positive (B) and negative (D) metabolites. Each point represents a metabolite substance, and the color indicates the metabolite density in that area. The darker the color, the larger the number of metabolites.
